# Multifunctional role of astrocytes as gatekeepers of neuronal energy supply

**DOI:** 10.3389/fncel.2013.00038

**Published:** 2013-04-10

**Authors:** Jillian L. Stobart, Christopher M. Anderson

**Affiliations:** ^1^Division of Neurodegenerative Disorders, Department of Pharmacology and Therapeutics, St. Boniface Hospital Research, University of ManitobaWinnipeg, MB, Canada; ^2^Department of Nuclear Medicine, Institute of Pharmacology and Toxicology, University of ZürichZürich, Switzerland

**Keywords:** astrocytes, brain oxidative metabolism, glutamate-glutamine shuttle, neurovascular coupling, Alzheimer's disease, ischemia, epilepsy

## Abstract

Dynamic adjustments to neuronal energy supply in response to synaptic activity are critical for neuronal function. Glial cells known as astrocytes have processes that ensheath most central synapses and express G-protein-coupled neurotransmitter receptors and transporters that respond to neuronal activity. Astrocytes also release substrates for neuronal oxidative phosphorylation and have processes that terminate on the surface of brain arterioles and can influence vascular smooth muscle tone and local blood flow. Membrane receptor or transporter-mediated effects of glutamate represent a convergence point of astrocyte influence on neuronal bioenergetics. Astrocytic glutamate uptake drives glycolysis and subsequent shuttling of lactate from astrocytes to neurons for oxidative metabolism. Astrocytes also convert synaptically reclaimed glutamate to glutamine, which is returned to neurons for glutamate salvage or oxidation. Finally, astrocytes store brain energy currency in the form of glycogen, which can be mobilized to produce lactate for neuronal oxidative phosphorylation in response to glutamatergic neurotransmission. These mechanisms couple synaptically driven astrocytic responses to glutamate with release of energy substrates back to neurons to match demand with supply. In addition, astrocytes directly influence the tone of penetrating brain arterioles in response to glutamatergic neurotransmission, coordinating dynamic regulation of local blood flow. We will describe the role of astrocytes in neurometabolic and neurovascular coupling in detail and discuss, in turn, how astrocyte dysfunction may contribute to neuronal bioenergetic deficit and neurodegeneration. Understanding the role of astrocytes as a hub for neurometabolic and neurovascular coupling mechanisms is a critical underpinning for therapeutic development in a broad range of neurodegenerative disorders characterized by chronic generalized brain ischemia and brain microvascular dysfunction.

## Introduction

The brain receives 10% of cardiac output but consumes 20% of total blood glucose and oxygen during cerebral activity to restore ion gradients after action potential conduction and neurotransmission (Magistretti et al., 1999; Magistretti, 2006). Large metabolic demand requires that brain blood flow remain constant despite variations in blood pressure (autoregulation) and that areas of high neuronal activity have correspondingly high metabolic rate and local blood supply (Magistretti, 2006). Astrocytes are multi-functional regulators of neurometabolic coupling that control uptake and release of neurotransmitters (Anderson and Swanson, 2000), influence local blood supply (Zonta et al., 2003; Mulligan and Macvicar, 2004; Takano et al., 2006; Gordon et al., 2008), and directly supply neurons with substrates for oxidative phosphorylation (Pellerin et al., 1998a).

Several characteristics of astrocytes confer suitability for sensing and satisfying neuronal metabolic needs. Protoplasmic astrocytes are highly organized into nearly unique three-dimensional domains (Oberheim et al., 2006) with limited overlap (Ogata and Kosaka, 2002). This feature places astrocytes non-randomly in virtually all central nervous system (CNS) 3D space, which is an ideal anatomical scenario for cells engaging in regional brain activity monitoring and/or corresponding nutritive distribution. Astrocyte process extensions from the soma define domain extremities and extensively ensheath central synapses (Ventura and Harris, 1999) producing a synaptic structure referred to as the “tripartite synapse” (Araque et al., 1999; Oberheim et al., 2006), in which astrocyte processes are located in close enough proximity to communicating nerve terminals that they receive neurotransmitter input. Astrocyte processes also envelop parenchymal brain arterioles and capillaries in unique spatial domains, extending terminal structures known as endfeet that are directly in contact with the vascular basal lamina (Simard et al., 2003; Oberheim et al., 2006). Endfeet express surface proteins, such as glucose transporters, for uptake of energy substrates from the endothelium (Kacem et al., 1998), and are capable of releasing transmitters that influence local blood flow (Simard et al., 2003; Zonta et al., 2003; Mulligan and Macvicar, 2004; Metea and Newman, 2006; Gordon et al., 2008). Astrocytes are therefore also uniquely positioned for bidirectional communication across the blood-brain barrier, as well as being participants in synaptic transmission. In addition, single hippocampal or cortical astrocytes are in contact with up to 600 dendrites (Halassa et al., 2007) and over 100,000 synapses (Bushong et al., 2002), and extend multiple processes to blood vessels (McCaslin et al., 2011). This provides a single-cell linkage between the locus of neuronal activity and sites that can leverage additional energy supply in an arrangement known as the neurovascular unit. This anatomy provides an astrocyte-mediated communication link for energy substrate transfer between blood supply and synaptic terminals (Tsacopoulos and Magistretti, 1996; Simard et al., 2003; Rouach et al., 2008).

Protoplasmic astrocytes also form a functional syncytium, where distal processes are connected by connexin gap junctions permitting diffusion of ions and metabolites between neighboring astrocytes (Giaume and McCarthy, 1996; Scemes et al., 1998). This creates a conduit for intercellular communication and flow of metabolites, but also allows intracellular communication through autocellular junctions between processes of the same cell (Wolff et al., 1998; Rouach et al., 2002). Connexin proteins also form hemichannels, which do not connect to adjacent cells, but allow release of small molecules from the cytoplasm into the extracellular space (Contreras et al., 2002; Rouach et al., 2002; Ye et al., 2003). This network of gap junctions is central to astrocyte function and control of brain metabolism, facilitating communication and movement of molecules within and around astrocyte domains.

Peri-synaptic or vascular astrocyte distributions would not be functionally relevant without mechanisms for receiving input. Astrocytes achieve this by expressing numerous types of neurotransmitter receptors that initiate electrically silent activation of astrocytes by enhancing intracellular Ca^2+^ levels. These broad receptor categories are coupled to G-proteins and activate a wide array of intracellular second messenger pathways, including inositol trisphosphate production and release of Ca^2+^ into the cytoplasm from endoplasmic reticulum stores (Sheppard et al., 1997; Idestrup and Salter, 1998). This permits astrocytes to respond to synaptic transmission through elevated cytosolic Ca^2+^. Astrocyte GPCR receptors involved in Ca^2+^ signaling cover a diverse range of neurotransmitters such as GABA_*B*_ receptors (Kang et al., 1998; Bettler et al., 2004; Meier et al., 2008), acetylcholine muscarinic receptors (Takata et al., 2011; Navarrete et al., 2012), α-adrenergic receptors (Duffy and Macvicar, 1995; Bekar et al., 2008), H1 histamine receptors (Shelton and McCarthy, 2000), endocannabinoid receptors (Navarrete and Araque, 2008, 2010), purinergic P2Y receptors binding adenine nucleotides (Guthrie et al., 1999), and metabotropic glutamate receptors (mGluRs) (Porter and McCarthy, 1996; Perea and Araque, 2007). Many papers implicate mGluR5 as a major activator of astrocyte Ca^2+^ (Bezzi et al., 1998; Zonta et al., 2003; Takano et al., 2006; Gordon et al., 2008; Liu et al., 2011), however, there is recent work suggesting mGluR5 expression decreases with age and does not stimulate Ca^2+^ signals in adult cortical and hippocampal astrocytes (Sun et al., 2013). More work on this is required before a consensus can be reached.

Ca^2+^ elevations may represent the fulcrum of a multi-faceted repertoire of potential astrocyte responses to sensory input. There is broad consensus that increased astrocytic intracellular Ca^2+^ triggers release of gliotransmitters such as glutamate, ATP, and D-serine (Bezzi et al., 2004; Mothet et al., 2005; Jourdain et al., 2007). Gliotransmitters, in turn, can affect synaptic activity (Parpura et al., 1994; Araque et al., 1999; Panatier et al., 2006; Henneberger et al., 2010; Sasaki et al., 2011; Fossat et al., 2012), produce constriction or dilation of local blood supply vessels (Zonta et al., 2003; Mulligan and Macvicar, 2004; Takano et al., 2006; Gordon et al., 2008) or have an autocrine effect to amplify Ca^2+^ signals (Suadicani et al., 2006). Additionally, elevation of Ca^2+^ in a single astrocyte is capable of initiating a similar response in surrounding astrocytes in a regenerative wave-like fashion. This process is primarily dependent on connexin 43 (Scemes et al., 1998; Blomstrand et al., 1999; Haas et al., 2006; Gosejacob et al., 2011) and release of extracellular gliotransmitters, including ATP (Hassinger et al., 1996; Guthrie et al., 1999) and may mediate fast, long-distance intercellular communication between astrocytes (Scemes and Giaume, 2006). It is important to note that recent data challenge the view that astrocyte Ca^2+^ modulates neuronal activity (Petravicz et al., 2008; Agulhon et al., 2010; Nedergaard and Verkhratsky, 2012) or even that adult astrocytes express Ca^2+^-mobilizing metabotropic glutamate receptors shown previously to be critical for synaptic effects of astrocytes (Sun et al., 2013). These findings are fueling debate about the functional roles of astrocytic Ca^2+^ responses in adult animals *in vivo*. Finer spatial resolution of astrocytic Ca^2+^ levels may reveal that local responses are limited to process microdomains and not necessarily the cell soma (Shigetomi et al., 2010, 2012; Di Castro et al., 2011), which could partially explain apparent discrepancies. Regional differences in astrocytic physiology and developmental changes in astrocytic expression of neurotransmitter receptors may also be factors. Systematic attention to animal age, brain regions imaged and spatial resolution of astrocyte Ca^2+^ imaging *in vivo* will greatly help resolve these issues.

Architectural organization, neurotransmitter receptor expression, and gliotransmitter release are features enabling astrocytes to be prime regulators of synaptic environment and transmission (Araque et al., 1999; Anderson and Swanson, 2000; Henneberger and Rusakov, 2010), neurovascular coupling (Zonta et al., 2003; Mulligan and Macvicar, 2004; Takano et al., 2006; Gordon et al., 2008), blood-brain barrier function (Ballabh et al., 2004) and carbon source shuttling to neurons in high demand periods (Pellerin et al., 1998a; Rouach et al., 2008). We will discuss the influence of astrocytes on the synaptic environment and cerebral bioenergetics, including how astrocytes handle glutamate, supply neurons with oxidative energy substrates and store glycogen. Mechanisms by which astrocytes couple glutamatergic neurotransmission with neuronal energy metabolism and blood flow regulation will also be discussed. Finally, we will survey astrocyte dysfunction in brain diseases and injuries, including ischemic stroke, epilepsy, and Alzheimer's Disease.

## Astrocytes control cerebral glutamate levels

Glutamate is quantitatively the dominant excitatory CNS neurotransmitter (Fonnum, 1984). Unregulated synaptic glutamate levels, however, can cause neuronal excitatory cell death in multiple diseases (Dong et al., 2009). Therefore, regulation of synaptic glutamate is crucial. Under normal conditions, glutamate balance in the neuropil is tightly controlled by astrocytes. Astrocytic processes enveloping glutamatergic synapses express active amino acid transport proteins that are the main route of extracellular glutamate removal (Rothstein et al., 1994; Danbolt, 2001). The primary glutamate transporters are Na^+^/glutamate co-transporters of the SLC gene family, termed excitatory amino acid transporter 1 and 2 (EAAT1 and 2) in human tissue (Shashidharan et al., 1994) or glutamate transporter-1 (GLT-1) and L-glutamate/L-aspartate transporter (GLAST) in rodents (Pines et al., 1992; Storck et al., 1992). These proteins rely on the Na^+^ electrochemical gradient, maintained by Na^+^/K^+^ ATPase activity, to co-transport 1 glutamate molecule and 3 Na^+^ ions. Glutamate uptake is energetically expensive, as ATP is consumed by Na^+^/K^+^ ATPases, but with sufficient energy supply, perisynaptic astrocyte processes prevent excitotoxic accumulation of glutamate in the neuropil. A Na^+^-independent, glutamate/cystine antiporter is also expressed by astrocytes but this is considered a secondary mechanism of glutamate uptake as these transporters primarily conduct cystine (Cho and Bannai, 1990).

Neurons may also take up glutamate through EAAT3 (EAAC1 in rodents) (He et al., 2000; Chen and Swanson, 2003), EAAT4 (Furuta et al., 1997; Nagao et al., 1997; Jackson et al., 2001), or EAAT5 (Arriza et al., 1997); however, the expression and localization of these transporters vary throughout the brain. For example, EAAT5 is mainly located in the retina (Arriza et al., 1997) and EAAC1 and EAAT4 are found on extrasynaptic neuronal membranes, particularly in the cerebellum, and are believed to modulate glutamate release and post-synaptic excitation (Tong and Jahr, 1994; Overstreet et al., 1999). EAAT3 also readily takes up cysteine (Chen and Swanson, 2003), which is used for glutathione production, suggesting EAAT3 has a central role in neuronal antioxidant defense (Aoyama et al., 2006).

**Figure 1:**
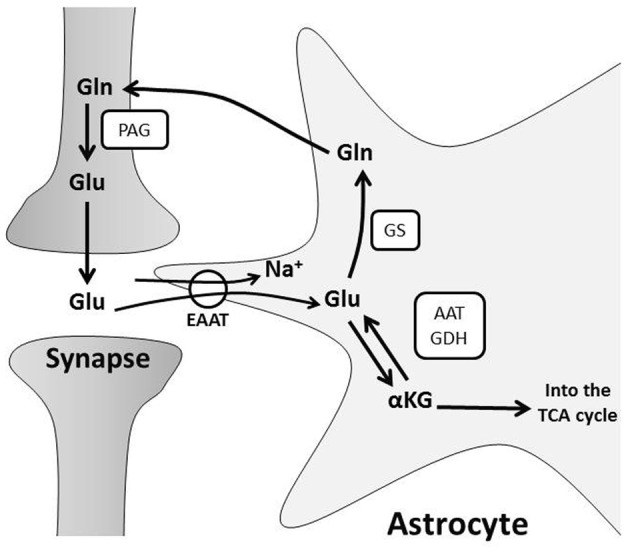
**The glutamate-glutamine cycle.** Glutamate (Glu) from pre-synaptic neurons stimulates post-synaptic neurons, and the signal is terminated by uptake of Glu from the synaptic cleft into astrocytes. Glu is primarily transported into astrocytes through Na^+^-dependent excitatory amino acid transporters, EAATs. This disrupts the astrocyte Na^+^ gradient and energy is consumed by the Na^+^/K^+^ ATPase to restore ionic concentrations. Glu is converted to: (a) glutamine (Gln) via glutamine synthase (GS) or (b) alpha-ketoglutarate (α-KG) by glutamate dehydrogenase (GDH) or aspartate aminotransferase (AAT) for subsequent oxidative metabolism in the TCA cycle. Gln is shuttled to neurons for glutamate production by phosphate-activated glutaminase (PAG) and the resulting Glu is repackaged in vesicles for further synaptic release.

Once synaptic glutamate enters astrocytes, one-third is used as a substrate for oxidative metabolism (Schousboe et al., 1993; Hertz and Zielke, 2004; Hertz et al., 2007). Glutamate can be converted to α-ketoglutarate by glutamate dehydrogenase or aspartate aminotransferase to replenish components of the tricarboxylic acid (TCA) cycle (Faff-Michalak and Albrecht, 1993; McKenna et al., 2006a). An additional portion of salvaged glutamate is recycled for neurotransmission through a process known as the glutamate-glutamine shuttle (Figure 1). Glutamate is converted to glutamine by astrocytic glutamine synthase (Martinez-Hernandez et al., 1977). Glutamine is then transported from the astrocytic cytoplasm by system N transporters and removed from the extracellular space by neuronal system A neutral amino acid transporters (Chaudhry et al., 2002). Neuronal glutamine is converted back to glutamate by phosphate-activated glutaminase (Kvamme et al., 2000) and repackaged into vesicles (Fremeau et al., 2004) for synaptic release (McKenna, 2007). This shuttle process is vital for proper synaptic glutamate release because neurons do not express enzymes for *de novo* synthesis of glutamate, so neuronal glutamate is entirely derived from astrocyte glutamine or α-ketoglutarate (Yu et al., 1983; Shank et al., 1985). Astrocytes produce *de novo* glutamate or glutamine from glucose via pyruvate conversion to oxaloacetate by pyruvate carboxylase (Yu et al., 1983; Hertz, 2011).

The glutamate-glutamine cycle not only drives neurotransmitter recycling, but also influences brain metabolism. Astrocytes metabolize glutamate to TCA cycle intermediates (Schousboe et al., 1993; Hertz and Zielke, 2004; Hertz et al., 2007), which diminishes the glutamate pool, and may drive astrocytic glucose consumption, ATP production and *de novo* glutamate synthesis (Hertz, 2011). Neurons also utilize glutamine and/or glutamate as energy substrates during glucose deprivation *in vitro* (Peng et al., 2007) or ischemia *in vivo* (Pascual et al., 1998). They similarly use glutamine or glutamate to replenish intermediates of the TCA cycle during metabolism of other substrates *in vitro* (Shokati et al., 2005). These observations suggest that the glutamate-glutamine shuttle impacts neuronal metabolism. Glutamate uptake by cultured astrocytes also correlates with increased glycolysis and lactate production (Pellerin and Magistretti, 1994). This is a separate mechanism of glutamate-driven astrocyte-neuron metabolic coupling that will be discussed below.

## Astrocyte lactate fuels neuronal metabolism

Synaptic glutamate is a direct signal of neuronal activity and, therefore, of metabolic demand. Astrocytes surveying synaptic activity respond with elevated glucose utilization, glycolysis (Pellerin and Magistretti, 1994; Cholet et al., 2001), and lactate production (Pellerin and Magistretti, 1994; Schurr et al., 1999; Voutsinos-Porche et al., 2003; Caesar et al., 2008). Enhanced astrocytic metabolism is thought to result from intracellular Na^+^ accumulation associated with Na^+^/glutamate co-transport (Voutsinos-Porche et al., 2003; Langer and Rose, 2009). This elevates ATP consumption by Na^+^/K^+^ ATPase activity resulting in increased glucose uptake, enhanced glycolytic rate, and lactate generation (Pellerin and Magistretti, 1994; Chatton et al., 2000; Loaiza et al., 2003; Porras et al., 2008). Intercellular Na^+^ waves are also generated throughout the astrocyte syncytium, elevating glucose uptake, and metabolism in neighboring astrocytes as well (Bernardinelli et al., 2004; Scemes and Giaume, 2006). Furthermore, K^+^ released during neurotransmission is taken up by astrocytes, which stimulates glycolysis and lactate export (Bittner et al., 2011; Ruminot et al., 2011).

**Figure 2:**
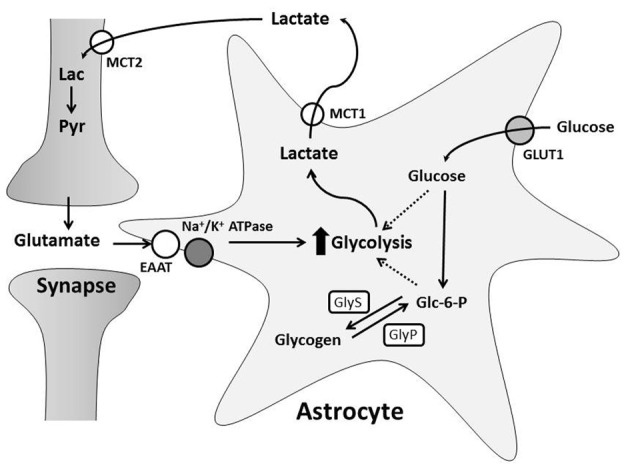
**The Astrocyte to Neuron Lactate Shuttle Hypothesis.** Free glucose is taken up by astrocytes through GLUT1 transporters and converted to glucose-6-phosphate (Glc-6-P). Glc-6-P is stored as glycogen synthesized by glycogen synthase (GlyS). During greater energy demand, glycogenolysis, mediated by glycogen phosphorylase (GlyP), creates Glc-6-P for glycolysis. Synaptic transmission induces astrocyte glycolysis and lactate production through glutamate uptake. This increases glucose consumption and/or glycogen breakdown in astrocytes. Astrocyte lactate is transported into the extracellular space by MCT1 and taken up through MCT2 by neurons. Neurons can convert lactate (Lac) to pyruvate (Pyr) for oxidative phosphorylation.

Glutamatergic neurotransmission increases both neuronal and astrocytic energy consumption, but the primary neuronal energetic substrate during normal and pathological conditions has been debated. One hypothesis is that neurons and astrocytes utilize systemically delivered glucose and oxygen from the extracellular space for metabolism by oxidative phosphorylation (Chih and Roberts, 2003). The second hypothesis proposes astrocytes convert glucose to lactate in an activity-dependent, glutamate-mediated manner for delivery to neurons (Pellerin and Magistretti, 1994; Pellerin et al., 1998a; Magistretti and Pellerin, 1999). This is known as the astrocyte-neuron lactate shuttle hypothesis (ANLSH) and suggests lactate is more than a potentially damaging final metabolite of anaerobic glycolysis (Figure 2; Kasischke, 2008).

In light of the ANLSH, a large body of literature pertaining to production and neuronal use of lactate has accumulated over the last 20 years. Several points have been made. First, there is a correlation between synaptic activity and extracellular lactate concentrations. At rest, the extracellular space around neurons and astrocytes has a homogenous concentration of lactate and glucose (Simpson et al., 2007; Barros and Deitmer, 2010). Extracellular lactate decreases slightly during short periods of brain activation *in vivo* (Hu and Wilson, 1997; Mangia et al., 2003), possibly because neurons are utilizing lactate for oxidative metabolism (Kasischke et al., 2004). However, extracellular lactate rapidly rises as neuronal stimulation continues for longer periods (Prichard et al., 1991; Mangia et al., 2007). Oxygen levels remain unchanged, suggesting brain activation stimulates aerobic glycolysis (Hu and Wilson, 1997). Second, *in vitro* studies demonstrate that glutamate induces glucose transporter (GLUT1) activity and uptake rates in astrocytes (Loaiza et al., 2003), while inhibiting neuronal glucose transporter activity (Porras et al., 2004). This suggests glutamatergic transmission may increase astrocyte glucose availability and reduce neuronal glucose metabolism. Third, lactate can support neuronal survival. In rodent brain slices, inhibition of lactate transport and glycolysis during exposure to glutamate caused a permanent loss of neuronal function (Schurr et al., 1999), while addition of lactate maintained synaptic activity in the absence of glucose (Schurr et al., 1988; Fowler, 1993; Izumi et al., 1997), preventing neurotoxicity (Schurr et al., 1997; Maus et al., 1999; Cater et al., 2001). Fourth, neurons express protein machinery necessary for lactate metabolism. Lactate metabolism is mediated by lactate dehydrogenase (LDH), which reversibly converts pyruvate to lactate with oxidation of NADH to NAD^+^ (Tsacopoulos and Magistretti, 1996). Several different LDH isoforms are located in the brain; LDH1 is the main isoform in neurons, while LDH1 and LDH5 are found in astrocytes (Bittar et al., 1996; Tsacopoulos and Magistretti, 1996). Lactate *consumption* is favored by neuronal LDH1, which promotes conversion of lactate to pyruvate (Bittar et al., 1996). In contrast, there is evidence that astrocytes favor *production* of lactate (Walz and Mukerji, 1988; Peng et al., 1994), likely due to the properties of LDH5, which has a higher affinity for pyruvate than lactate (Bittar et al., 1996). Lactate is transported between the intracellular and extracellular spaces by monocarboxylate transporters (MCT). MCT are symporters that co-transport lactate anions with H^+^, suggesting lactate transport is driven by pH (Schneider et al., 1993; Barros and Deitmer, 2010). The distribution of MCTs in the brain is heterogeneous: MCT1, MCT2, and MCT4 are expressed by astrocytes, while neurons express predominately MCT2 (Broer et al., 1997; Gerhart et al., 1998; Pellerin et al., 1998b; Bergersen et al., 2001; Pierre et al., 2002). MCT2 co-localizes with post-synaptic density proteins in dendritic spines and has the highest affinity for lactate of all MCTs (Bergersen et al., 2001). Together, MCT2 and LDH1 provide neurons with lactate protein machinery ideally suited to remove and metabolize lactate from active synapses. Lastly, a recent study found that an important activator of glycolysis, 6-phosphofructo-2-kinase/fructose 2,6-bisphosphatase isoform 3 (Pfkfb3), is continually degraded in neurons (Herrero-Mendez et al., 2009). This suggests glucose metabolism is shifted toward the pentose phosphate pathway and antioxidant production, and that neurons have a low glycolytic rate, necessitating utilization of lactate for aerobic respiration.

There is also a correlation between changes in neuronal and astrocytic redox states and lactate transport and metabolism that may support the ANLSH (Hirrlinger and Dringen, 2010). During glycolysis, cytosolic NADH is produced and must be oxidized back to NAD^+^ in order for glycolysis to continue. NAD^+^ is replenished by lactate production or redox shuttle systems (glycerol-3-phosphate and malate-aspartate shuttle, MAS) which transfer reducing equivalents to the mitochondrial electron transport complexes. While the level of involvement of the glycerol-3-phoshpate shuttle in neuronal NAD^+^ homeostasis is not clear (Cammer and Zimmerman, 1982; Waagepetersen et al., 2001; Nguyen et al., 2003), the MAS is important for regenerating NAD^+^ for glutamate neurotransmitter renewal and energy metabolism (Palaiologos et al., 1988; McKenna et al., 2006b). Inhibition of the MAS-elevated cytosolic NADH, disrupting the redox balance and limiting lactate consumption (McKenna et al., 2006b) without affecting glucose metabolism in synaptic terminals (McKenna et al., 1993). Moreover, disruption of the MAS in malate-aspartate carrier (aralar) deletion mice resulted in impaired neuronal development (Gomez-Galan et al., 2012), reduced dopamine levels (Llorente-Folch et al., 2013), and hypomyelination (Ramos et al., 2011), indicating this pathway affects neuronal function in a profound way. There is no clear evidence that this is related to lactate metabolism, however.

The role of the malate-aspartate shuttle in astrocytes is currently debated. Several groups show that astrocytes express low levels of aralar (Ramos et al., 2003; Berkich et al., 2007), which limits MAS activity and requires elevated lactate production to replenish cytosolic NAD^+^ (Schurr, 2006; Lemire et al., 2008). In support, astrocyte lactate-to-pyruvate ratios were unchanged in aralar knockout mice, compared to wild type controls (Pardo et al., 2011). In contrast, a recent paper suggests adult cultured astrocytes express aralar and the MAS could be functional (Li et al., 2012). This makes the importance of MAS in astrocytes difficult to determine at this point. Nevertheless, there is a clear correlation between cytosolic redox states and lactate production in astrocytes. In cultured astrocytes, inhibition of oxidative phosphorylation (which elevates cytosolic NADH) increases lactate production and regenerates NAD^+^ (Dringen et al., 1993). High levels of NADH also influence transcription factors, including Clock and NPAS2, which activate LDH1 expression in astrocytes (Rutter et al., 2001), further potentiating lactate production. Again, extracellular lactate increases during longer periods of neuronal stimulation (~10 s), and a corresponding elevation of astrocytic cytosolic NADH concentrations is also observed (Kasischke et al., 2004). This means astrocytes may replenish extracellular lactate pools for shuttling to neurons during prolonged activation (Pellerin et al., 1998a; Magistretti and Pellerin, 1999; Magistretti et al., 1999; Bouzier-Sore et al., 2002).

Mathematical modeling has been used to approximate the flux of energy metabolites between neurons and astrocytes based on known mass balances and enzyme/transporter kinetics, with the goal of linking *in vitro, in vivo*, and functional imaging results. Several models have recently been presented, but with varying results. One model describes energy substrates (lactate, glucose, pyruvate), oxygen, and NADH concentrations within the neuronal and astrocyte energy compartments, while also considering the subcellular compartments (cytosol and mitochondria) (Aubert et al., 2007), glutamate transport, and astrocyte glycogen (Cloutier et al., 2009). Results from this model support the ANLSH (Aubert et al., 2007; Cloutier et al., 2009) and the flow of lactate from astrocytes to neurons. The second model focuses on glucose and lactate transport between the blood-brain barrier, neurons, and astrocytes and suggests that neurons primarily metabolize glucose and export lactate (Simpson et al., 2007; Mangia et al., 2009). This supports a neuron to astrocyte lactate shuttle hypothesis (NALSH) (Simpson et al., 2007; Mangia et al., 2009). A third model attempts to combine metabolism rates and concentrations from the first model with transporter kinetics and metabolite diffusion equations from the second model and the results also support a neuron to astrocyte lactate shuttle (Dinuzzo et al., 2010). While the outcomes and design of these mathematical models continue to be debated, each model succeeds in raising questions to be addressed by future experiments. Most notably, there is evidence that neurons can utilize lactate as an energy source during periods of activation, but the question remains: do astrocytes produce lactate for neuronal consumption? Clearly, neurons and astrocytes produce and utilize lactate differently based on the expression profiles and properties of LDH and MCT isoforms, but due to experimental limitations of lactate detection, it is not possible to distinguish lactate producers from the cell type that utilizes lactate, or if these roles change depending on region or activity (Barros and Deitmer, 2010). Measurement of radiotracer kinetics *in vivo* suggest neurons consume lactate during activation (Wyss et al., 2011), and further *in vivo* studies may elucidate the complex flux of brain metabolites. In particular, experiments involving awake animals may more accurately reflect brain metabolic states, as anesthetics are known to decrease metabolic rates (Alkire et al., 1995, 1997, 1999). It would also be beneficial to directly visualize *in vivo* glucose and lactate levels (possibly via fluorescent sensors for glucose or lactate) to determine metabolite concentrations in different cell populations in various brain regions during activation (Barros et al., 2013; San Martin et al., 2013).

Astrocyte lactate is not only a potential energy substrate, but also acts as a signaling molecule in other brain bioenergetic processes, including blood flow regulation (discussed in detail later) (Gordon et al., 2008), blood glucose sensing (Lam et al., 2005, 2007), and sodium sensing in the subfornical organ (SFO; Shimizu et al., 2007). Brain lactate is involved in a brain-liver signaling axis. Hypothalamic arcuate nuclei projections to the brainstem signal to vagal hepatic efferents (Schwartz et al., 2000; Grill et al., 2002) to regulate blood glucose levels (Lam et al., 2005) and insulin signaling (Pocai et al., 2005). Elevated blood glucose leads to increased glial glucose uptake (Chari et al., 2011) and lactate production in the rodent hypothalamus (Lam et al., 2005). Lactate is transported into hypothalamic neurons for conversion to pyruvate. This process is required to activate neuronal ATP-sensitive K^+^ channels (K_ATP_) (Lam et al., 2005), and K^+^ flux that induces hyperpolarization and reduces firing (Pocai et al., 2005). Resulting hepatic vagal stimulation (Pocai et al., 2005) reduces gluconeogenesis and glycogenolysis rates (Lam et al., 2005; Pocai et al., 2005), leading to secretion of very-low density lipoprotein (Lam et al., 2007) and reduced expression of hepatic enzymes for endogenous glucose production, including glucose-6-phosphatase (Lam et al., 2005; Pocai et al., 2005; Kishore et al., 2011). This provides a lactate-mediated brain-liver negative feedback axis (Lam et al., 2007), which has implications in obesity and hepatic insulin resistance. In particular, hypothalamic glial GLUT1 expression and glucose uptake are decreased during hyperglycemia in rodents *in vivo*, and this could form the basis of blood glucose dysregulation in diabetes (Chari et al., 2011). Also, intracerebroventricular injection of lactate decreased blood glucose levels in animal models of uncontrolled diabetes and diet-induced insulin resistance, independent of insulin signaling (Chari et al., 2008), which suggests that hypothalamic lactate could be a future therapeutic target.

In the SFO of the brain periventricular region, lactate influences salt intake behavior and blood Na^+^ sensing (Shimizu et al., 2007). Glial cells of the SFO express atypical sodium (Na_*x*_) channels (Hiyama et al., 2004), which have a concentration-sensitive, extracellular sodium threshold of 150 mM (Hiyama et al., 2002). SFO glial Na_*x*_ channels interact with Na^+^/K^+^ ATPase and progressive Na^+^ influx upon elevated extracellular Na^+^ triggers anaerobic glucose metabolism and lactate production (Shimizu et al., 2007). Lactate and Na_*x*_ channels mediate salt-intake behavior, since Na_*x*_-knockout mice continue to ingest salt when dehydrated (Hiyama et al., 2004) and they have reduced SFO lactate concentrations compared to wild type animals (Shimizu et al., 2007). Salt-intake behavior is reduced when glial lactate stimulates inhibitory neurons in the SFO by a MCT-dependent mechanism (Shimizu et al., 2007). This mechanism may also involve inhibition of K_ATP_ channels by lactate-induced ATP production (Shimizu et al., 2007); however, further experiments are required to determine the involvement of these channels in the pathway. These studies of the role of lactate in glucose and sodium sensing and food intake behaviors indicate an exciting new role for lactate as a signaling molecule to neurons and suggest the importance of lactate in the brain may be underestimated.

## Astrocyte glycogen production fuels neuronal metabolism

Glycogen is the main cellular storage depot of glucose in mammals (Brown and Ransom, 2007). When glucose is in excess of immediate energy requirements, it can be stored as glycogen; glycogen is mobilized to glucose when glucose levels cannot meet energy demands (Brown and Ransom, 2007). Astrocytes are the main glycogen repository in the adult brain (Phelps, 1972; Koizumi, 1974). Astrocytes express both glycogen synthase (GlyS, for glycogen formation) and glycogen phosphorylase (GlyP, for glycogen degradation) (Pellegri et al., 1996) and glycogen stores are primarily located in regions of high synaptic density, such as gray matter (Phelps, 1972; Sagar et al., 1987). Astrocyte glycogen is critical for maintaining neuronal survival and synaptic activity during hypoglycemia *in vitro* (Swanson and Choi, 1993) and *in vivo* in cortex, hippocampus (Suh et al., 2007) and optic nerve (Wender et al., 2000). Similarly, during periods of increased brain activity and local glucose depletion, astrocyte glycogen stores can be rapidly degraded to provide a temporary energy supply (Shulman et al., 2001; Brown et al., 2003, 2005).

Glycogen cycling occurs when astrocytes acquire glucose through the glucose transporter, GLUT1, and rapidly phosphorylate it to glucose 6-phosphate in the first steps of glycolysis, preventing it from leaving the cell (Vannucci et al., 1997). Glucose-6-phosphate can be converted to glycogen through a process catalyzed by GlyS (Figure 2). GlyS exists in both an inactive phosphorylated form and an active dephosphorylated form. Astrocyte glycogen formation is therefore regulated by enzymes that dephosphorylate and activate GlyS, most notably protein phosphatase 1 which acts via the regulatory subunit Protein Targeting to Glycogen (PTG) (Allaman et al., 2000). Expression of PTG is stimulated by numerous molecules such as vasoactive intestinal peptide, norepinephrine, and adenosine, which increase glycogen production (Sorg and Magistretti, 1992; Allaman et al., 2000). Similarly, GlyP can be regulated by phosphorylase kinase, which converts GlyP from its inactive form to its active, phosphorylated form (Brown and Ransom, 2007). GlyP is only expressed in astrocytes, solidifying the specialization of these cells in glycogen utilization. Glycogenolysis results in glucose-6-phosphate, which can be metabolized within astrocytes to lactate (Dringen and Hamprecht, 1993; Tekkok et al., 2005) or free glucose (Ghosh et al., 2005). This suggests astrocyte glycogen-derived substrates can be supplied to other brain cells for oxidative metabolism.

The astrocytic glycogen reservoir is dynamic under normal brain activity and euglycemic conditions (Brown et al., 2005), and is influenced by glutamatergic neurotransmission and uptake. Glutamate triggers glycogenolysis to meet the energy demand of the glutamate-glutamine cycle and Na^+^ gradient restoration, in addition to the mechanisms proposed in the ANLSH (Shulman et al., 2001). Glycogenolysis fuels glutamate uptake by enhancing active transport-mediated recovery from the extracellular space, since inhibition of glycogenolysis-elevated extracellular glutamate concentrations (Sickmann et al., 2009; Schousboe et al., 2010). Glycogenolysis also facilitates *de novo* synthesis of glutamate and glutamine (Sickmann et al., 2005; Gibbs et al., 2006, 2007). Therefore, astrocyte glycogen is important for supporting the energetic needs of glutamatergic neurotransmission.

Recent studies have found glycogen-derived lactate is central to higher cognitive function and memory formation (Gibbs et al., 2006; Newman et al., 2011; Suzuki et al., 2011). In day old chicks, a bead discrimination learning task for memory consolidation was impaired after inhibition of glycogenolysis (Gibbs et al., 2006, 2007) or injection of poorly metabolized D-lactate (which competes with L-lactate for transport) (Gibbs and Hertz, 2008). An *in vivo* study of rats during an inhibitory avoidance test found learning-induced glycogenolysis and lactate release that was important for long-term memory formation (Suzuki et al., 2011). This was determined by administering inhibitors of glycogen phosphorylation or knocking down expression of MCT1/4 or MCT2, which induced amnesia. Inhibition of glycogen phosphorylation also reduced long-term potentiation (LTP), which was rescued by lactate injection (Suzuki et al., 2011). In another rat study during a spontaneous alternation task to assess spatial working short-term memory, lactate concentrations increased during the task and inhibition of glycogenolysis and lactate transport decreased task success (Newman et al., 2011). These results suggest astrocyte glycogenolysis and lactate transport to neurons is required for working memory processing and long-term memory consolidation.

While debate over the primary neuronal energy source will likely continue, it is clear that there is situational activity-dependent regulation of neuronal metabolism by astrocytes involving glycogen cycling, lactate production, and the glutamate-glutamine shuttle. This metabolic coupling of astrocytes and neurons appears to be important for higher cognitive function.

## Astrocytes mediate vasomotor responses based on tissue energy demand

Neuronal activity is tightly coupled to increased local blood flow by neurovascular coupling in a response termed functional hyperemia. Neurovascular coupling is a complex, multi-modal response involving numerous identified signaling pathways and resulting in vasodilation of penetrating arterioles upstream of regions with enhanced of activity, and vasoconstriction in regions with abundant substrate supply and lower activity (Devor et al., 2007). The net effect of this response is to enhance glucose and oxygen delivery from blood to meet neuronal and glial energy demands.

Astrocytic spatial architecture permits relay of signals from synapses to penetrating arterioles and capillaries. As part of the multi-faceted response of astrocytes to increased neuronal activity, synaptic neurotransmission triggers elevated intracellular astrocyte Ca^2+^ through diverse receptor types including GABA_*B*_ receptors (Kang et al., 1998; Bettler et al., 2004; Meier et al., 2008), acetylcholine muscarinic receptors (Takata et al., 2011; Navarrete et al., 2012), α-adrenergic receptors (Duffy and Macvicar, 1995; Bekar et al., 2008), H1 histamine receptors (Shelton and McCarthy, 2000), endocannabinoid receptors (Navarrete and Araque, 2008, 2010), mGluR_5_ (Zonta et al., 2003), and P2Y receptors (Simard et al., 2003). Astrocyte cytosolic Ca^2+^ elevations (Simard et al., 2003; Zonta et al., 2003; Filosa et al., 2004; Schummers et al., 2008), and inositol-3-phosphate signaling (Straub et al., 2006) are central to neurovascular coupling, stimulating release of vasoactive compounds that dilate or constrict neighboring arterioles (Zonta et al., 2003; Mulligan and Macvicar, 2004; Metea and Newman, 2006; Takano et al., 2006; Gordon et al., 2008). The polarity (i.e., constriction vs. dilation) of these vascular responses involves multiple pathways, discussed in later sections.

### Arachidonic acid metabolites

**Figure 3:**
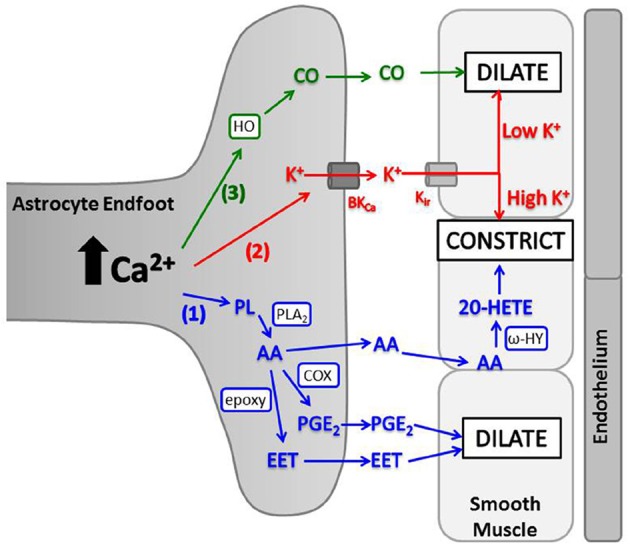
**Astrocyte intracellular Ca^2+^ elevations trigger release of vasoactive molecules. (1)** PLA_2_ is activated by Ca^2+^ and converts phospholipids (PL) to AA. AA is metabolized in astrocyte endfeet to PGE_2_ (by COX) or EET [by cytochrome P450 epoxygenase (epoxy)] which dilate arterioles, or AA can diffuse to smooth muscle where ω-hydroxylase (ω-HY) converts it to 20-HETE and causes constriction. **(2)** K^+^ is released from astrocyte endfeet through BK_Ca_, and the amount of K^+^ released is directly proportional to astrocyte Ca^2+^ level. K^+^ is taken up into smooth muscle through K_ir_ and causes dilation at low concentrations and constriction at high concentrations. **(3)** HO is activated by Ca^2+^ and produces CO, which diffuses to smooth muscle and triggers dilation.

Elevated astrocyte cytosolic Ca^2+^ stimulates activity of phospholipase A_2_ (PLA_2_), which hydrolyzes phospholipids to produce arachidonic acid (AA) (Mulligan and Macvicar, 2004; Sun et al., 2005). AA metabolism by several enzymes produces different molecules with variable vascular effects (Figure 3). In brain slices and *in vivo*, a non-selective cyclooxygenase (COX) or COX-1 inhibitor blocked arteriolar vasodilation after astrocyte Ca^2+^ stimulations, suggesting AA is metabolized by astrocyte COX-1 to prostaglandin E_2_ (PGE_2_) (Zonta et al., 2003; Takano et al., 2006; Gordon et al., 2008). In cortical astrocytes and retinal glia, AA is also metabolized by cytochrome P450 epoxygenase to vasodilator, epoxyeicosatrienoic acids (EETs) (Peng et al., 2002; Metea and Newman, 2006; Liu et al., 2011). Both PGE_2_ and EETs open smooth muscle large conductance Ca^2+^-sensitive K^+^ (BK_Ca_) channels, triggering hyperpolarization and decreased voltage-gated calcium channel (VGCC) activity (Gebremedhin et al., 1992; Miura and Gutterman, 1998; Higashimori et al., 2010). EETs also indirectly stimulate BK_Ca_ channels by increasing Ca^2+^ sparks (Earley et al., 2005). AA metabolism can also cause vasoconstriction. AA can diffuse to smooth muscle cells and be rapidly metabolized by ω-hydroxylase (another cytochrome P450 enzyme) to produce 20-hydroxyeicosatetraenoic acid (20-HETE) (Mulligan and Macvicar, 2004; Metea and Newman, 2006). 20-HETE causes smooth muscle contraction by inhibiting vascular BK_Ca_ K^+^ channels, leading to depolarization and increased Ca^2+^ entry through VGCC.

At first, these opposing effects of astrocyte AA metabolism on vascular lumen diameter represented a confusing dichotomy in the field. However, the last 5 years have brought some mechanistic clarity showing that the directional control of AA metabolism is finely controlled by metabolic need and nitric oxide (NO). In brain slices and retinal preparations equilibrated with 95–100% oxygen, elevated astrocyte Ca^2+^ led to vasoconstriction mediated by 20-HETE production (Mulligan and Macvicar, 2004; Gordon et al., 2008; Mishra et al., 2011). However, in brain slices and retinal preparations treated with 20% oxygen, astrocyte Ca^2+^ elevations caused vasodilation induced by PGE_2_ produced from COX-1 activity (Gordon et al., 2008; Mishra et al., 2011). Vasodilation induced by direct astrocyte Ca^2+^ stimulation *in vivo* was also mediated by COX-1 (Takano et al., 2006). Interestingly, part of the mechanism for dictating response directionality appears to be related to lactate production by astrocytes, revealing another critical role for lactate alongside the ANLSH. At 20% oxygen, astrocytes oxidize glucose and produce lactate (Gordon et al., 2008). Astrocyte endfeet express a prostaglandin-lactate transporter that exchanges intracellular lactate for extracellular PGE_2_ (Chan et al., 2002). Thus, at 20% oxygen, increased extracellular lactate from astrocyte glycolysis inhibits the prostaglandin-lactate transporter, resulting in elevated extracellular PGE_2_ and vasodilation (Gordon et al., 2008). Current consensus suggests astrocytes maintain vascular tone equilibrium (between vasodilation and vasoconstriction) under physiological conditions. When synaptic activity is minimal and oxygen consumption is low, vasoconstriction by 20-HETE is favored because PGE_2_ is taken up rapidly through prostaglandin-lactate transporters. During periods of elevated activity, oxygen is depleted and lactate is released from astrocytes, leading to inhibition of the prostaglandin-lactate transporter, more extracellular PGE_2_, and vasodilation. This mechanism couples cerebral blood flow regulation and the ANLSH, since astrocyte lactate production may act as a neuronal energy source and signaling molecule to increase blood flow.

While astrocytes may “sense” the oxygen content in their local environment by producing variable lactate levels *in vitro* (Gordon et al., 2008), the relevance of this mechanism *in vivo* is not clear. *In vivo*, oxygen levels may not influence neurovascular coupling like they do *in vitro* preparations (Lindauer et al., 2010; Mishra et al., 2011). In *ex vivo* retinal preparations, for example, while incubation with 100% oxygen increases tissue partial pressure of oxygen (pO_2_) 16-fold, administering 100% oxygen to anesthetized rats only modestly elevates retinal pO_2_ (Mishra et al., 2011). Consequently, retinal neurovascular coupling favors vasodilation under normoxic and hyperoxic conditions *in vivo*, in contrast to vasoconstriction *in vitro* under high pO_2_. In addition, physiologic cerebral oxygen levels are between 12 and 38 mmHg (Jamieson and Vandenbrenk, 1963; Metzger et al., 1971; O'Hara et al., 2005), suggesting 20-HETE synthesis, which is dependent on binding of molecular oxygen as a cofactor and has a K_m_O_2_ (Michaelis constant for oxygen) of 60–70 mmHg (Harder et al., 1996), is low in normoxia. Conversely, production of dilatory prostaglandins and EETs, both with K_m_O_2_ ≤ 10 mmHg (Harder et al., 1996; Juranek et al., 1999), would be favored at physiologic oxygen. This suggests that the effect of oxygen on the kinetics of AA metabolism may be sufficient to dictate vascular response polarity as observed *in vitro*; however, the influence of oxygen on responses *in vivo* may favor dilation and requires further investigation.

The role of NO in functional hyperemia further complicates neurovascular signaling, as NO also modulates AA metabolism (Metea and Newman, 2006). Traditionally, NO has been considered a direct vasodilator, stimulating vascular smooth muscle guanylyl cyclase leading to activation of K^+^ channels and hyperpolarization (Ignarro et al., 1999). However, NO can also inhibit cytochrome P450 enzymes, such as ω-hydroxylase, thereby reducing 20-HETE production (Alonso-Galicia et al., 1998, 1999), or cytochrome P450 epoxygenase, mitigating EET production (Udosen et al., 2003). Additionally, NO weakly activates COX-1, while suppressing COX-2 (Fujimoto et al., 2004), which may affect prostaglandin levels. Overall, cerebral vasodilation by NO likely involves both smooth muscle effects and inhibition of 20-HETE production, thereby favoring lumen expansion by prostaglandins and EETs (Sun et al., 2005; Attwell et al., 2010). The opposite effect of NO on AA metabolism, that is inhibition of dilatory metabolism in deference to 20-HETE, may also occur at elevated tissue oxygen *in vitro*. In retinal preparations maintained in 95% O_2_, NO-enhanced constriction produced by glial activation in a manner thought to result from inhibition of EET formation (Metea and Newman, 2006). There is evidence that all NOS isoforms (i.e., nNOS, eNOS, or iNOS) could be involved in 20-HETE modulation. Reduction of vasodilation *in vivo* by an nNOS inhibitor was reversed by 20-HETE inhibition, suggesting neuronal NO inhibits 20-HETE production in live animals (Liu et al., 2008). Recent evidence from brain slices also indicates that eNOS permits dilation through suppression of 20-HETE synthesis (Stobart et al., 2013). iNOS is more likely to be involved in neurovascular coupling during pathological conditions. Inhibition of iNOS, which is elevated in retinal glia of diabetic animal models, rescued functional hyperemia, possibly by attenuating EET production (Mishra and Newman, 2010).

### Potassium

Extracellular K^+^ is generated by working neurons and is an effective vasodilator, giving it suitable properties as a neurovascular coupling mediator. Astrocytes have long been known to regulate neuronal membrane potential by removing synaptic K^+^ (Amedee et al., 1997; Kofuji and Newman, 2004), and astrocytes express inwardly rectifying K_ir_4.1 K^+^ channels (Sontheimer and Waxman, 1993; Sontheimer, 1994) and large conductance Ca^2+^-sensitive K^+^ channels (BK_Ca_) on vascular endfeet (Price et al., 2002) as a potential egress route for vasodilatory K^+^. In retinal preparations, it was suggested vasodilation can be triggered via K^+^ efflux through glial endfoot K_ir_4.1 channels in response to neurotransmission (Newman et al., 1984; Paulson and Newman, 1987), but studies of K_ir_4.1 knockout mice failed to support this idea (Metea et al., 2007). A second mechanism was proposed involving Ca^2+^-dependent astrocyte BK_Ca_ channels (Filosa et al., 2006). The idea is that astrocytic Ca^2+^ increases lead to BK_Ca_ channel activation, K^+^ release, and smooth muscle relaxation. Moderate astrocytic Ca^2+^ increases indeed triggered BK_Ca_ channel-induced dilation of neighboring arterioles, but larger astrocyte Ca^2+^ signals produced greater BK_Ca_ channel opening, higher astrocyte K^+^ release and vasoconstriction (Figure 3) (Girouard et al., 2010). This polarity was dictated by a threshold extracellular K^+^ ([K^+^]_o_) concentration of 20 mM. Lower than this threshold, conductance of smooth muscle inward rectifying K_ir_2.1 K^+^ channels (Bradley et al., 1999) was enhanced, causing hyperpolarization, reduced VGCC activity, and vascular smooth muscle relaxation (Girouard et al., 2010). In contrast, [K^+^]_o_ larger than 20 mM caused smooth muscle depolarization, increasing VGCC conductance, and vasoconstriction (Knot et al., 1996; Knot and Nelson, 1998). This represents another potential mechanism of activity-dependent vasodilation mediated by astrocytes. Moreover, it is another mechanism by which astrocytes could theoretically select for dilation or constriction based on magnitude of K^+^ release (Dunn and Nelson, 2010).

AA metabolite and K^+^ signaling occur in parallel to regulate cerebral blood flow (Filosa et al., 2006) and may interact since AA metabolites also affect smooth muscle ion conductance. In renal arteries, PGE_2_ can induce smooth muscle BK_Ca_ channel- mediated dilation through EP2 or EP4 prostanoid receptors (Zhang et al., 2005), but this mechanism has not been tested in cerebral arteries. Also, astrocyte BK_Ca_ channel activity is increased by EETs (Higashimori et al., 2010), suggesting AA metabolites can modulate K^+^ release into the perivascular space, but the vascular implications of this interaction have not been studied.

### Carbon monoxide

Carbon monoxide (CO) is produced by heme oxygenase (HO) and can have vasoactive effects. On a cellular level, CO can relax vascular smooth muscle by increasing coupling between smooth muscle BK_Ca_ channels and local Ca^2+^ transients, similar to EET activity (Figure 3; Jaggar et al., 2002; Wu et al., 2002; Xi et al., 2010). CO-mediated vasorelaxation has been observed in peripheral tissues such as liver (Suematsu et al., 1994, 1995) and carotid arteries (Brian et al., 1994), but cerebrovascular results are varied (Brian et al., 1994; Leffler et al., 1999; Ishikawa et al., 2005; Leffler et al., 2006a; Li et al., 2008; Xi et al., 2010, 2011; Morikawa et al., 2012). Brain arteries from rabbits and dogs demonstrated no response to CO (Brian et al., 1994), while arteries from rats and piglets dilated in response to CO (Leffler et al., 1999; Jaggar et al., 2002; Holt et al., 2007; Li et al., 2008; Xi et al., 2010, 2011) or constricted based on CO-induced inhibition of NO dilation pathways (Ishikawa et al., 2005). In piglet studies, glutamate-induced vasodilation was mediated by CO, as HO inhibitors blocked lumen diameter increases in isolated arteries (Fiumana et al., 2003) and pial arteries *in vivo* (Leffler et al., 1999; Robinson et al., 2002). Glutamate stimulates endothelium-dependent dilation through CO production from endothelial and smooth muscle cells (Fiumana et al., 2003; Leffler et al., 2003), but also induces CO production in astrocyte endfeet (Leffler et al., 2006b; Parfenova et al., 2012) by Ca^2+^ and calmodulin-dependent activation of HO (Xi et al., 2011). This astrocyte-specific response can reportedly mediate vasodilation *in vivo* (Li et al., 2008) indicating CO is another diffusible, vasoactive molecule, released upon astrocytic activation by neurotransmission. Astrocyte CO production and dilation of piglet pial arteries *in vivo* can be enhanced by adenosine diphosphate (Kanu and Leffler, 2009), NO (Barkoudah et al., 2004; Leffler et al., 2005a,b), AA and PGE_2_ (Kanu et al., 2006; Kanu and Leffler, 2011), suggesting an interaction between other dilatory mechanisms and HO activity. A study of adult rat pial arteries *in vivo* indicated CO-induced cerebral vasoconstriction by inhibiting NO production (Ishikawa et al., 2005), and similar results were observed in piglets, but after prolonged exposure to CO (Knecht et al., 2010; Leffler et al., 2011). Therefore, there may be a polarity to CO-mediated cerebrovascular effects, akin to similar effects seen with AA metabolism and K^+^ effects.

In summary, astrocytes are not only important for regulating synaptic environments and the supply of energy metabolites to neurons, but they are also central to the regulation of neurovascular coupling by releasing several molecules, including AA metabolites, K^+^, and CO, in response to synaptic transmission. We are only just beginning to understand how these pathways work in concert to fine-tune regulation of cerebral blood flow.

## Astrocyte control of cerebral bioenergetics can contribute to disease

Multiple brain diseases and injuries are associated with aberrant energy metabolism, dysfunctional glutamate cycling by astrocytes, and altered neurovascular coupling. Here, we discuss the major bioenergetic changes and astrocyte dysfunction in Alzheimer's disease (AD), cerebral ischemia, and epilepsy.

### Alzheimer's disease

AD is the most common form of dementia, characterized by declining cognitive performance and memory (McKhann et al., 1984). AD pathology is characterized by two types of lesions—amyloid-β (Aβ) plaques, consisting of insoluble, extracellular deposits of Aβ peptide fibrils, and neurofibrillary tangles, composed of intracellular neuronal deposits of hyperphosphorylated and crosslinked tau protein (Merz et al., 1983; Braak and Braak, 1988). Aβ peptides are linked to synaptic dysfunction, activation of microglia and astrocytes, and oxidative stress, but the precise contribution of plaque formation to disease pathogenesis remains controversial (Fuller et al., 2009).

During AD, astrocytes undergo morphological changes, related to proximity of Aβ deposits. In dementia patients and transgenic mice, extensive reactive gliosis appears near Aβ plaques (Rodriguez et al., 2009; Simpson et al., 2010), while astrocytes farther away display dystrophic changes such as decreased complexity, surface area, and volume of cell processes (Senitz et al., 1995; Rodriguez et al., 2009). In many cases, abnormal glial morphology occurs early in disease on-set before amyloid deposition is apparent (Scheff et al., 2007; Rodriguez et al., 2009). Astrocyte dystrophy and reactive astrogliosis may greatly impair astrocytic modulation of synaptic environments and neuronal metabolism, exacerbating AD progression (Fuller et al., 2009; Steele and Robinson, 2012). For example, brain glucose metabolism is diminished in pre-clinical patients (Mosconi et al., 2008) and cerebral glucose uptake in transgenic AD mice (Merlini et al., 2011) and AD patients (Alexander et al., 2002) is significantly reduced, often before Aβ plaques or neurofibrillary tangles are detected (Small et al., 2000). Glycogen-derived lactate is important for memory formation in healthy brain (Gibbs et al., 2006; Newman et al., 2011; Suzuki et al., 2011), and dysfunction of this pathway could contribute to AD pathogenesis. Transgenic AD mice demonstrate decreased brain lactate release during neuronal stimulation (Merlini et al., 2011). In day-old chicks treated with Aβ 1–42 peptide, memory consolidation was rescued upon injection of energy substrates, such as acetate, a substrate oxidized specifically by astrocytes (Gibbs et al., 2009). This suggests Aβ may damage astrocyte glycolysis and lactate production, reducing brain metabolism, and impairing memory.

The astrocyte glutamate-glutamine shuttle is also altered during AD. Expression of astrocyte glutamate transporter, EAAT2, is reduced in both transgenic mice and dementia patients, suggesting astrocytes take up less synaptic glutamate (Li et al., 1997; Masliah et al., 2000; Simpson et al., 2010). Also, both glutamine synthetase activity (Smith et al., 1991) and the concentration of glutamine in cerebrospinal fluid is reduced in AD patients (Csernansky et al., 1996; Jimenez-Jimenez et al., 1998). The confluence of these events results in a dysregulation of glutamate homeostasis and reduced transfer of glutamine to neurons from astrocytes. Neurons in AD brains aberrantly express astrocyte proteins, including the amino acid transporter, EAAT1 (Scott et al., 2002), and glutamine synthetase (Robinson, 2000), possibly in an attempt to normalize glutamate handling and limit excitotoxicity. Neuronal expression of EAAT1 is correlated with neurofibrillary tangle formation (Scott et al., 2002), while glutamine synthetase expression corresponds with plaque formation (Robinson, 2000). Since these enzymes and transporters are critical for glutamate uptake and the glutamate-glutamine shuttle, such dramatic changes in cellular distribution suggest profound astrocyte dysfunction and impaired glutamate handling during AD. In combination with reduced energy metabolism, this may greatly affect neuronal viability and synaptic transmission (Rodriguez et al., 2009).

Impaired vascular reactivity, reduced neurovascular coupling, and diminished resting blood flow are all associated with AD (Mentis et al., 1996; Warkentin and Passant, 1997; Niwa et al., 2000, 2001; Iadecola, 2004), and could be attributed to astrocytes and hemodynamic dysfunction. Cultured astrocytes treated with Aβ peptides (1–42 and 25–35) (Abramov et al., 2003; Chow et al., 2010) and *in vivo* astrocytes from transgenic AD mice exhibit increased frequency of spontaneous, focal intracellular Ca^2+^ responses not coupled with neuronal activity (Takano et al., 2007; Kuchibhotla et al., 2009). Intercellular Ca^2+^ waves between astrocytes were also increased in frequency and amplitude in both cultured cells and *in vivo* (Haughey and Mattson, 2003; Kuchibhotla et al., 2009). Furthermore, Aβ 40-peptide accumulates in blood vessel walls (Selkoe and Schenk, 2003; Agyare et al., 2012) causing endothelial cell deformity, smooth muscle deterioration (Farkas and Luiten, 2001; Merlini et al., 2011), and pericyte toxicity (Wilhelmus et al., 2007). This is linked to reduced free NO and vasoconstriction (Thomas et al., 1996; Niwa et al., 2001), and suggests that Aβ accumulation may alter the functional neurovascular unit. The concentration of reactive oxygen species (ROS) also increases in AD transgenic mice (Park et al., 2004), which are known to reduce production of vaso-active molecules, as observed *in vitro* (Fleming, 2004; Sun et al., 2008). Thus, dysfunctional neurovascular coupling during AD could be caused by altered astrocyte Ca^2+^ signaling, increased ROS, and gross vascular abnormalities, which change normal intrinsic vascular tone. Astrocyte dysfunction appears to be central to AD initiation and progression, and these cells have now become future therapeutic targets (Fuller et al., 2009).

### Cerebral ischemia

During cerebral ischemia, blood flow is restricted by cortical or subcortical occlusion, chronically impaired vascular reactivity or cardiac arrest. Bioenergetic failure results (Hertz, 2008) in a cytotoxic cascade characterized by lactate and proton acidification (Silver et al., 1997) and ROS generation, (Abramov et al., 2007), inhibition of Na^+^/K^+^ ATPases, membrane depolarization (Silver et al., 1997) and elevation of extracellular glutamate due to depolarization-induced vesicular release and non-vesicular egress mechanisms. This initiates further membrane depolarization, mitochondrial damage, excitotoxicity, and neuronal death (Schild et al., 2003; Brookes et al., 2004; Nicholls, 2004; Nicholls et al., 2007). Neurons are very sensitive to this chain reaction, while astrocytes are more resistant because they can increase their glycolytic rate (Walz and Mukerji, 1990) or utilize alternate energy substrates for ATP production (Edmond et al., 1987; Hertz, 2003; Hertz and Hertz, 2003). Astrocytes also exploit glutathione stores to limit ROS damage (Juurlink, 1997). In early ischemic stages, astrocytes may help ailing neurons, but prolonged ischemic stress damages astrocytes, which may contribute to neuronal demise (Rossi et al., 2007). As described below, astrocytes affect neuronal survival and metabolism during ischemia through glutamate handling, lactate shuttling, and glycogen breakdown, and the transport of metabolites through gap junctions.

During ischemia, neuronal ionic gradients are disrupted by Na^+^/K^+^ ATPase inhibition, elevating extracellular glutamate concentrations (Bosley et al., 1983; Goldberg et al., 1988; Hillered et al., 1989). In early stages, astrocytes take up and accumulate intracellular glutamate (Hertz et al., 1998; Voloboueva et al., 2007) in an attempt to balance the extracellular environment, but they continue to shuttle glutamine to neurons, facilitating additional glutamate release (Haberg et al., 2001). Prolonged ischemia disrupts the glutamate-glutamine cycle (Gorovits et al., 1997) due to depleted ATP levels, accumulation of intracellular Na^+^ and reversal of GLT1 and GLAST to cause facilitated extrusion of glutamate (Anderson and Swanson, 2000; Phillis et al., 2000; Bonde et al., 2003). Furthermore, astrocytes swell and release yet more glutamate through volume-regulated anion channels (Kimelberg et al., 1990). These observations have generated interest in astrocyte glutamate handling as a potential ischemic therapeutic target, since upregulated expression or activity of glutamate transporters or inhibition of volume-regulated anion channels may decrease glutamate excitotoxicity (Rossi et al., 2007).

Progression of neuronal death during ischemia is dependent on availability of energy substrates. Experimental inhibition of lactate transporters (MCTs) during ischemia exacerbates neuronal death and astrocytes display increased conversion of glycogen to lactate (via glucose-6-phosphate) during this time (Brown et al., 2005; Tekkok et al., 2005; Suh et al., 2007), suggesting lactate and glycogen are important for maintaining ATP levels and neuronal survival. Lactate can also diffuse through astrocyte gap junctions (Rouach et al., 2008), which remain open during ischemia (Cotrina et al., 1998), facilitating the beneficial flux of lactate within the astrocytic network. As oxygen is depleted, astrocytes appear to be able to sustain neuronal function via anaerobic glycolysis (Rossi et al., 2007). However, there is a fine balance between benefit and injury and eventually lactate builds to concentrations which induce acidosis and cellular damage (Li and Siesjo, 1997). Further experimental testing is required to determine the role of astrocyte glycogen during ischemia. Brain regions with higher than normal glycogen concentrations are more resistant to ischemic damage (Swanson et al., 1989), and increasing glycogen stores in cultured astrocytes reduces neuronal death during glucose deprivation (Swanson and Choi, 1993). Protective effects *in vivo* may also be enhanced by increasing glycogen stores, either through inhibition of GlyP (Suh et al., 2007) or by elevating glycogen synthase activity (Rossi et al., 2007). In cell culture models of ischemia, propagation of signals and metabolites through the glial network is increased through hemichannels (Contreras et al., 2002). This may exacerbate tissue damage as increased hemichannel activity allows Na^+^ and Ca^2+^ to diffuse into astrocytes, while glutamate flows out, furthering excitotoxicity (Ye et al., 2003). Also, in astrocyte cultures, glutathione (an important astrocytic antioxidant) is lost over time through hemichannels, limiting ROS protection (Rana and Dringen, 2007). While both hemichannels and gap junctions respond to ischemic signals, they are difficult *in vivo* therapeutic targets as both are inhibited by the same antagonists, obscuring potential benefits (Rossi et al., 2007).

Reperfusion after ischemia is characterized by reduced blood flow (Leffler et al., 1989) due to disruption of the neurovascular unit via neuronal and vascular ischemic damage (Del Zoppo, 2010). Reduced neurovascular coupling exacerbates ischemic injury, which may increase infarct size. Blood flow is partly reduced because fibrin, activated platelets and/or leukocytes occlude capillaries and venules (Del Zoppo and Mabuchi, 2003). Evidence also suggests that AA metabolite (EETs and 20-HETE) signaling is altered during ischemia, which contributes to decreased blood flow and neurovascular coupling. Recent therapeutic studies have elevated EET levels using inhibitors of soluble epoxide hydrolase (sEH), an enzyme that degrades EETs (Imig and Hammock, 2009). sEH inhibitors are beneficial regardless of administration time, since infarct size is decreased in rodents when the drug is given chronically, shortly after the ischemic insult or during reperfusion (Dorrance et al., 2005; Zhang et al., 2007, 2008; Simpkins et al., 2009). EETs mediate this protection, as inhibition of CYP epoxygenase (the EET synthesis enzyme) prevents sEH benefits (Zhang et al., 2007, 2008). This protective mechanism increases astrocyte survival (Liu and Alkayed, 2005), elevates antiapoptotic factors (Simpkins et al., 2009) and increases neurovascular coupling (Zhang et al., 2007, 2008). Conversely, 20-HETE is elevated during ischemia (Tanaka et al., 2007), and inhibition of 20-HETE production is also neuroprotective in rodent models (Miyata et al., 2005; Poloyac et al., 2006; Tanaka et al., 2007; Dunn et al., 2008; Renic et al., 2009). Reduction of 20-HETE inhibits ROS production (Dunn et al., 2008), limits vasoconstriction and increases blood flow during reperfusion (Miyata et al., 2005; Dunn et al., 2008). Taken together, evidence suggests AA metabolite signaling is dysfunctional during and after cerebral ischemia, whereby EETs are decreased and 20-HETE is elevated. By inhibiting EET degradation and 20-HETE production, functional hyperemia can be restored, and these pathways make promising therapeutic targets.

Focal cerebral ischemia causes altered glutamate handling and lack of energy substrates, which triggers neuronal excitotoxicity, ATP depletion, and ROS production (Hertz, 2008). In early stages of ischemia, astrocytes are less susceptible to damage and may help protect neurons through glutamate uptake, glycogen hydrolysis to lactate for energy, and conduction of protective molecules through gap junctions. However, prolonged ischemia damages the neurovascular unit reducing blood flow and functional hyperemia during reperfusion. Current therapeutic targets are meant to promote astrocyte protection of neurons and help restore proper circulation after stroke.

### Epilepsy

Epilepsy is characterized by sudden, temporary synchronization of electrical charges in groups of neurons, which may manifest as seizures. The origins of this disorder are not completely understood (McCormick and Contreras, 2001; Scharfman, 2007), but neuronal hyperexcitability is believed to be caused by disequilibrium between glutamatergic and GABAergic neurotransmission, either by decreased inhibitory (GABA) circuits or excessive glutamatergic release (Dudek et al., 1999; Uhlhaas and Singer, 2006). Dysfunctional astrocyte glutamate-glutamine cycling is also involved (Tian et al., 2005), as astrocyte expression of EAAT2 is diminished in epilepsy patients (Proper et al., 2002; Fotheringham et al., 2007), and knock-down of glutamate transporters [EAAC1 (Sepkuty et al., 2002), GLT-1 (Tanaka et al., 1997), and GLAST (Watase et al., 1998)] in animal models exacerbates neuronal excitability. Also, glutamine synthetase expression is reduced by 40% in astrocytes of epilepsy patients, suggesting that glutamate degradation is greatly diminished (Eid et al., 2004). Therefore, dysfunctional glutamate metabolism in astrocytes could contribute to neuronal synchronization and hyperexcitability.

Ion homeostasis by astrocytes is altered during epilepsy. Particularly, both K_ir_ currents and aquaporin 4 expression are reduced, (D'Ambrosio, 2004; Eid et al., 2005) and this results in elevated extracellular K^+^, decreased water homeostasis, and reduced seizure thresholds (Binder and Steinhauser, 2006). Astrocytes also display elevated intracellular Ca^2+^ signals before and during seizure activity in rodents (Tian et al., 2005; Gomez-Gonzalo et al., 2010, 2011), which are mediated by mGluR and purinergic receptors, and may further exacerbate neuronal activation by triggering gliotransmission (Gomez-Gonzalo et al., 2010). Interestingly, common antiepileptic drugs, such as valproate and phenytoin, reduce astrocytic Ca^2+^ increases (Tian et al., 2005).

Cerebral bioenergetics are aberrantly regulated in epilepsy, but the precise changes remain unknown. Epilepsy patients display high levels of glucose uptake and hypermetabolism during seizures (Engel et al., 1983), and low levels of glucose uptake and hypometabolism between seizures (Engel et al., 1982). In animal models of epilepsy, astrocyte glycogen accumulates before the onset of seizures for possible conversion to neuronal energy substrates (Bernard-Helary et al., 2000). Glycolytic inhibitors, such as 2-deoxy-D-glucose, have antiepileptic properties (Garriga-Canut et al., 2006), suggesting glycolysis is necessary for neuronal hyperexcitability and synchronization. Also, glucose flux from blood vessels to neurons through astrocytic gap junctions can partially sustain epileptiform activity in brain slices (Rouach et al., 2008). However, connexin knockout mice experience spontaneous interictal bursts and neuronal hyperexcitability, which has been attributed to decreased buffering of extracellular K^+^ and glutamate (Wallraff et al., 2006; Cloix and Hevor, 2009; Pannasch et al., 2011; Bedner and Steinhauser, 2013). Gap junction trafficking is reportedly altered in epilepsy, possibly permitting elevated extracellular K^+^ and glutamate, but how this effects the flow of energy substrates remains unclear (Bedner and Steinhauser, 2013).

Epileptiform activity triggers increased blood flow and deoxygenates hemoglobin (Suh et al., 2006) to meet energy and oxygen demand of active neurons (Kuhl et al., 1980). However, hyperemia may not fully support neurons, since some studies suggest chronic epilepsy may cause ischemic-like tissue damage (Suh et al., 2006). A lag time was identified between astrocyte endfeet Ca^2+^ elevations and vasodilation of pre-constricted arterioles during synchronous bursts in rat brain slices treated with 95% oxygen, indicating astrocyte-independent neurovascular coupling mechanisms may be more prevalent in epilepsy (Gomez-Gonzalo et al., 2011). However, the cellular pathways influencing the hemodynamic response during epilepsy have not been investigated (Kovacs et al., 2012).

Astrocytes may play an important role in epilepsy, but it is unclear if they promote neuronal excitability, or merely sustain seizures and epileptogenesis. Several astrocyte functions are altered during epilepsy including glutamate-glutamine shuttle, ion homeostasis, and movement of metabolites, but the role of astrocytes in functional hyperemia during seizure activity is unknown. In the future, astrocyte glutamate uptake, blood flow control, or metabolism could be targeted to limit neuron excitability.

## Conclusion

Astrocytes were once considered the “glue” of the brain with little importance to brain function; however, they have emerged as modulators of brain bioenergetics, blood flow, and neuronal survival. Based on spatial orientation, gap junction connections, and complexity, astrocytes are well-situated to influence synaptic environments and function as “gatekeepers” of neuronal metabolism and blood flow. This involves complex, multi-modal mechanism where astrocytes “listen” to synaptic activity and respond through (a) glutamate uptake and recycling via the glutamate-glutamine cycle, (b) increased glycolysis and shuttling of metabolites to neurons for oxidative phosphorylation, and (c) elevated Ca^2+^ signaling and release of vasoactive molecules for blood flow control. These responses ensure astrocytes tightly couple neuronal metabolic need with enhanced supply. Furthermore, astrocyte dysfunction may contribute to aberrant neuronal metabolism and neurovascular coupling in disease and injury and these pathways are promising therapeutic targets.

### Conflict of interest statement

The authors declare that the research was conducted in the absence of any commercial or financial relationships that could be construed as a potential conflict of interest.
